# *OptiFit*: Computer-Vision-Based Smartphone Application to Measure the Foot from Images and 3D Scans

**DOI:** 10.3390/s22239554

**Published:** 2022-12-06

**Authors:** Riyad Bin Rafiq, Kazi Miftahul Hoque, Muhammad Ashad Kabir, Sayed Ahmed, Craig Laird

**Affiliations:** 1 Department of Computer Science and Engineering, University of North Texas, Denton, TX 76203-5017, USA; 2 Department of Computer Science and Engineering, Chittagong University of Engineering and Technology, Chottogram 4349, Bangladesh; 3 School of Computing, Mathematics and Engineering, Charles Sturt University, Bathurst, NSW 2795, Australia; 4 School of Health and Human Sciences, Southern Cross University, East Lismore, NSW 2480, Australia; 5 Foot Balance Technology Pty. Ltd., Westmead, NSW 2145, Australia; 6 EYM (Ease Your Motion) Pty. Ltd., Westmead, NSW 2145, Australia; 7 Walk Easy Pedorthics Pty. Ltd., Tamworth, NSW 2340, Australia

**Keywords:** foot measurement, computer vision, image processing, 3D scan, algorithm, smartphone app, custom footwear

## Abstract

The foot is a vital organ, as it stabilizes the impact forces between the human skeletal system and the ground. Hence, precise foot dimensions are essential not only for custom footwear design, but also for the clinical treatment of foot health. Most existing research on measuring foot dimensions depends on a heavy setup environment, which is costly and ineffective for daily use. In addition, there are several smartphone applications online, but they are not suitable for measuring the exact foot shape for custom footwear, both in clinical practice and public use. In this study, we designed and implemented computer-vision-based smartphone application *OptiFit* that provides the functionality to automatically measure the four essential dimensions (length, width, arch height, and instep girth) of a human foot from images and 3D scans. We present an instep girth measurement algorithm, and we used a pixel per metric algorithm for measurement; these algorithms were accordingly integrated with the application. Afterwards, we evaluated our application using 19 medical-grade silicon foot models (12 males and 7 females) from different age groups. Our experimental evaluation shows that *OptiFit* could measure the length, width, arch height, and instep girth with an accuracy of 95.23%, 96.54%, 89.14%, and 99.52%, respectively. A two-tailed paired *t*-test was conducted, and only the instep girth dimension showed a significant discrepancy between the manual measurement (MM) and the application-based measurement (AM). We developed a linear regression model to adjust the error. Further, we performed comparative analysis demonstrating that there were no significant errors between MM and AM, and the application offers satisfactory performance as a foot-measuring application. Unlike other applications, the iOS application we developed, *OptiFit*, fulfils the requirements to automatically measure the exact foot dimensions for individually fitted footwear. Therefore, the application can facilitate proper foot measurement and enhance awareness to prevent foot-related problems caused by inappropriate footwear.

## 1. Introduction

The foot is the foundation of the human body, as it supports and balances the entire structure of the body. Consequently, footwear should be designed in a manner that provides comfort and healthy effects to the user. Improper footwear is a major reason for discomfort, pain, and various foot-related problems [1,2]. Many people occasionally suffer from problems wearing regular-sized shoes. Moreover, patients with diabetes and athletes prefer custom shoes, as they better satisfy the fits [3,4]. Taking care of the feet should be a primary concern for everyone, as poor foot health can negatively impact our productivity and overall health [5,6]. Therefore, appropriate custom footwear with a good fit is necessary for foot health.

To produce custom footwear, foot measurements should be accurate, as they depend on the different dimensions of the foot. To tackle the problem of measuring the size of a foot, there are different ways to measure for best fitting. Manual measurement involves the use of a measuring tape and a sliding calliper  [7,8,9,10]. Moreover, a foot-measuring device called the Brannock device${}^{®}$ [11] has become quite popular and has been used for several years [12,13]. Recently, automatic measurement using laser-scanned data has become more popular for calculating foot dimensions [7,14]. However, existing approaches and technologies to measure feet are limited in several aspects and have some critical drawbacks. Foot measurement using a 3D scanner is too costly and time-consuming [7]. In contrast, manual measurement is not always accurate because its accuracy depends on the skills of the operator [15,16]. Additionally, it is not feasible for mass usage. Therefore, a faster and operator-independent solution with greater accuracy should be developed for public use.

A wide range of smartphone applications are available on app stores for measuring the foot dimensions [17]. However, existing applications lack consistency with the device architecture and primarily focus on specific measurements that cannot provide the entire structure of the foot to produce custom footwear [17]. There is no application that gives any information on how users can understand the accuracy of measurement with respect to their original dimensions.

Recently, computer-vision technologies have been applied to various real-world applications such as optical character recognition (OCR), machine inspection, object recognition, 3D model building, and medical imaging [18]. Moreover, computer-vision-based techniques have been utilized in foot-dimension measurement [19], 3D foot shape reconstruction [20,21], diabetic foot ulcer classification [22], and foot clearance measurement system development [23]. Thus, this approach can provide a solution for low-cost foot measurement that instantaneously calculates the foot dimensions.

In this paper, we present *OptiFit*, a computer-vision-based smartphone application to measure the foot shape for the optimized fitting of footwear. Our application can measure the length, width, arch height, and instep girth of a foot from images and a 3D mesh of a human foot. Our application also provides calibration accuracy that helps the user in quantifying the accuracy of measured dimensions. We evaluated *OptiFit* using 19 medical-grade silicon foot models that demonstrated up-to-the-mark results for all measurements.

This paper presents the following four major contributions:

1. We devised a pixel per metric algorithm to measure the length, width, and arch height of a foot, and propose an algorithm to calculate instep girth from a 3D foot model.
2. We developed a mathematical model to reduce the instep girth measurement error.
3. We designed and developed a smartphone application that is integrated with the above-mentioned algorithms to automatically measure four essential foot dimensions (length, width, arch height, and instep girth).
4. We evaluated the accuracy of our application using 19 medical-grade silicon foot models.

The remainder of the paper is organized as follows. Section 2 presents the background and related work on foot measurement. The methodology and the proposed algorithms for measuring the foot dimensions are described in Section 3. Section 4 presents the architecture, design, and implementation of the application. Section 5 reports the results of our experimental evaluation. Lastly, Section 6 concludes the paper and highlights the future work.

## 2. Background and Related Work

For footwear design, different foot dimensions, such as the length, width, height, and girth of feet, should be equal to those of the designed shoes [24,25,26,27]. An overview of our four foot dimensions is provided below.

**Figure 1:**
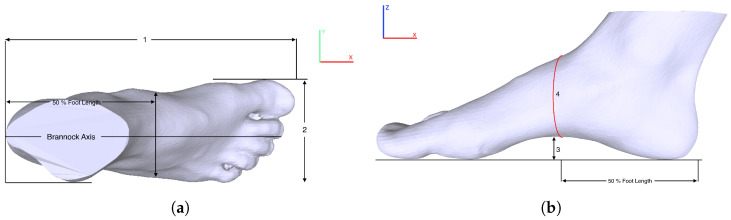
Foot dimensions. (**a**) Top view—foot length and width are marked as 1 and 2, respectively; (**b**) side view—arch height and instep girth are marked as 3 and 4, respectively.

Foot length is the distance along the Brannock axis (*x*-direction) from the pternion to the tip of the longest toe [7,28,29,30] (Mark 1 in Figure 1a). It is calculated as the distance between the minimal and maximal *x* values along the *x*-axis [7]:

(1) $$L_{FL}=\left|x_{min}-x_{max}\right|$$

Foot width is defined as the maximal horizontal breadth (*y*-direction) across the foot, perpendicular to the Brannock axis in the region in front of the most laterally prominent point on the fifth metatarsal head (Mark 2 in Figure 1a). It is calculated as the distance between the points with the minimal and maximal *y* values along the *y*-axis of the fore-foot [7]:

(2) $$W_{FW}=\left|y_{min}-y_{max}\right|$$

Arch height is established as the height between the bottom of the navicular tuberosity and the floor [31] (Mark 3 in Figure 1b). Arch height, along with other foot dimensions, provides distinctive features to compute an exact foot shape.

Instep girth is defined as the maximal circumference measured from the most plantar aspect of the foot to the most dorsal aspect of the foot, at the navicular level [24,32] (Mark 4 in Figure 1b). There are six important girth dimensions for customized shoes: ball girth, instep girth, long heel girth, short heel girth, ankle girth, and waist girth [24]. Among them, we focused only on the instep girth, as it provides an appropriate measurement of a foot along with the length, width, and arch height. In this study, instep girth was computed by selecting the middle point along the *x*-axis at first and then computing the circumference of points along the *yz* plane from the 3D mesh point cloud.

Different techniques have been applied to the field of foot measurement [8,9,28,33,34]. With the growth of technological development, laser scanners with diverse applications have been used in this field [14,35,36,37,38,39]. In laser technology, captured information is processed for known errors [40], and the slices of scanned data along the foot length were used for human foot modelling [41]. However, measurement accuracy would be affected if the subject moved their body during scanning [42,43]. The existing approach to the automatic extraction of foot dimensions is heavily dependent on 3D scanners. Several applications for 3D scanners were used in commercial, clinical, and research areas related to the human foot [44]. Liu et al. [15] recommended a technique for acquiring foot dimensions using a three-dimensional digitizing device. Witana et al. [7] proposed an automatic approach for foot measurement using 3D scanned data that was estimated through a comparison of the simulated measurements (SMs), the measurements obtained from the scanner software defined as the commercial program (CP), and the manual measurements (MM). In one study [45], a radial basis function (RBF) surface modelling technique was implemented for girth measurement, as the accuracy was laboriously subject to the density of the scanned 3D point cloud data. In [46], the authors experimented with three different alignment methods of foot scans and their effects on 10 different measurements. Foot dimensions were measured from a 3D foot model using an Intel SR300 camera in [47].

Apart from 3D scanners, many works used multicamera images on the basis of principal component analysis (PCA) [48,49,50]. Xiong et al. [51] presented a computer-aided design (CAD) system for designing custom footwear that automatically extracted the features of a foot, and their method worked basically in three main modules. In sequence, Xiong et al. [52] exhibited an advanced method of obtaining foot measurements from a 2D digital image; it was dependent on the hardware setup system. In [53], a foot-shape feature parameter measurement algorithm using a second-generation Kinect was implemented, and the authors measured foot length, foot width, metatarsal girth, and other foot parameters using a 3D foot depth image. Dickerson and Queen [54] suggested a custom-built, inexpensive foot posture measurement system (FPMS) that showed valid and accurate results compared to a 3D scanner.

Existing smartphone applications use the automated edge detection feature and they are prone to errors when the lighting condition is poor. In [55], the authors proposed a mobile solution using computer vision algorithms to extract six foot parameters. Additionally, other smartphone solutions used augmented reality and 3D scanning for foot dimension measurement [56,57]. MOBILE FOOT ID is an Android application that measures only foot length and width, and uses a paper sheet as a reference object. A smartphone application, Avatar3D, based on data-driven 3D construction technology, has shown more reliability than manual foot measurements did [58,59]. This data-driven approach used PCA to generate an arbitrary foot model, which was optimized after several iterations [60]. However, our method can construct a 3D foot model using the structure sensor or LiDAR while maintaining the individual characteristics of the foot. Moreover, in [61], the study assessing the reliability of the application lacked a comparison between the application-based measurements and the original foot dimensions. Only six apps (Nimco Professional Shoe Sizing, Fischer Scan-Fit, 3D Avatar Feet, SUNFeet, 3DSizeME, and Anodyne Scanner) provide detailed information on foot dimensions, and among them, 3DSizeME is notable because of its data sharability options and performance [17]. As the applications depend on specific measurements, there is no application that fulfils the criteria for calculating the exact shape of the foot. Table 1 summarizes and contrasts all the above-mentioned applications and how they differ from our developed *OptiFit*. To the best of our knowledge, there is no application that measures four-foot dimensions like ours.

The above-discussed strategies from different studies and applications were purposed to produce accurate measurements of distinct foot dimensions for designing custom footwear. Most of the mentioned studies focused on a heavy setup environment, which is inefficient for daily use. In addition, the measurements can be influenced by the skill level of a measurer with the instruments. The laser beam can be diverged due to the presence of dust particles or high levels of ambient light. This may introduce noise, and affect the accuracy of scanned point data [35,62]. Thus, errors are usually found in scanned data. Compared to the above studies, our smartphone iOS solution, *OptiFit*, measures the four foot dimensions more conveniently (i.e., without requiring a complicated setup) and accurately. We also considered the feature of calibration accuracy, which is displayed to help the users in understanding the reliability of the application. Therefore, the application assists pedorthists in measuring the foot for custom footwear. The users could also determine their foot measurements not only for custom footwear, but also for online footwear purchases without their physical presence.

## 3. Methods

In this study, we developed two algorithms and integrated them into our *OptiFit* iOS application to automatically calculate the foot dimensions. We devised a pixel-per-metric algorithm that utilizes a unit metric to determine the distance between two points from an image (Section 3.1). We used this algorithm to calculate the length, width, and arch height of a foot from a 2D image using our application. Furthermore, we developed the instep girth measurement algorithm (Section 3.2) to compute the circumference of the middle region of a foot from the 3D point cloud data. We performed a paired *t*-test to evaluate the measurement error of *OptiFit*. Only the instep girth measurement showed a noticeable error. We further developed a mathematical model to adjust the error.

### 3.1. Measuring Length, Width, and Arch Height

We measured the distance (length, width, or arch height) between any two points in an image on the basis of a reference object (also captured in the image). The reference object could be a coin or any circular object. Our pixel-per-metric algorithm (Algorithm 1) takes an image as input from a mobile camera and performs Hough circle transformation [63] to detect the circular (reference) object. Then, the total number of pixels along the diameter of the circular object is calculated. Subsequently, the total number of pixels is divided by the diameter of the reference object (a known constant value in millimetres, inches, or any other unit) to compute the pixel-per-metric value (for example, 6 pixels/mm) for that image. Given any two points in the image, the distance between them can be calculated by taking the total number of pixels between those two points and dividing it by the pixel per metric value.
**Algorithm 1** Pixel Per Metric1:*Input:* Bitmap from camera *image*, original diameter of circular object *diameterInMM*2:*Output:* PixelPerMetric3:*Initialization: diameterInPixels* = 04:diameterInPixels← Hough Circle Transform (*image*) ▹ Retrieved from circular object in image5:pixelPerMetric←diameterInPixels/diameterInMM▹*diameterInMM* is retrieved from device storage

### 3.2. Measuring Instep Girth

The instep girth measurement algorithm (Algorithm 2) cuts a slice from a 3D foot point cloud data along the *x*-axis.
**Algorithm 2** Instep girth measurement1:*Input:* Foot mesh point-cloud *Points[1...n]*2:*Output:* Instep girth3:*Initialization:* Filter dimension, *range* = 0.5; sorted points according to *x* coordinates, *sortedPoints* = []; girth measurement value, *girth* = 0, points that lie on the convex hull, *hullPoints* = []; 3D points converted to 2D, R = []; Convex hull points size, hullSize = 0, middle point of feet, middle = 0;4:sortedPoints←sort(Points)▹ Sort all points by comparing *x* coordinates5:middle←(sortedPoints[1].x+sortedPoints[n].x)/2▹ Determine the middle point of the foot6:**for**i←1 to *n* **do**7:    **if** Points[i].x>(middle−range) && Points[i].x<(middle+range) **then**8:        filteredPoints←Points[i]     ▹ Filter out points that are outside of range9:    **end if**10:**end for**11:R←Reduce(filteredPoints)▹ Remove the *x* coordinates from filtered points and make it 2D12:hullPoints←*graham’sScan* (*R*)13:hullSize←*length* (hullPoints)14:**for**i←1 to hullSize **do**15:    distance← *euclideanDistance* (hullPoints[i], hullPoints[i+1])16:    girth = girth + distance17:**end for**18:girth = girth + *euclideanDistance* (hullPoints[hullSize], hullPoints[1])

This is performed by sorting the point cloud data on the basis of *x* coordinates (line 4 in Algorithm 2). Then, the middle point of the foot is determined by taking the maximal and minimal values from the sorted array and dividing them by 2 (line 5). A range of 5 mm was taken, and all the points beyond that range from the middle point were filtered out (lines 6–10). Later, the filtered points were transformed from 3D into 2D by removing their respective *x* coordinates (line 11). Graham’s scan algorithm [64] (Algorithm 3) calculates the convex hull from the filtered points (line 12).
**Algorithm 3** Graham’s Scan1:*Input:* Filtered 2D points *Points[1...n]*2:*Output:* Boundary points of instep girth3:*Initialization:* Points sorted according to *y* coordinates *sortedPoints* = [], Points sorted by polar angle *polarPoints* = [], Empty Stack *S* = ∅; bottom most point, bottomPoint = nil;4:sortedPoints←sort(Points)   ▹ Sort all the points by comparing their *y* coordinates5:bottomPoint←sortedPoints[1]6:polarPoints←sort(sortedPoints[2...n])   ▹ Sort the points by comparing their polar angle respect to bottomPoint7:PUSH(bottomPoint,S)8:PUSH(polarPoints[1],S)9:PUSH(polarPoints[2],S)10:**for**i←3 to n−1 **do**11:    **while** the angle formed by NEXT-TO-TOP (S), TOP (S), polarPoints[i] makes a non left turn **do**12:        POP(S)13:    **end while**14:    PUSH(polarPoints[i],S)15:**end for**16:return *S*

**Figure 2:**
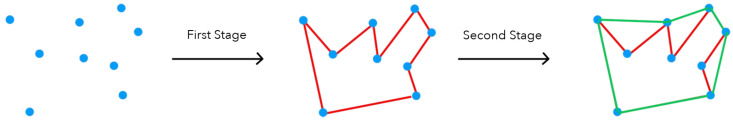
Two sequential stages for Graham’s scan algorithm.

Graham’s algorithm uses a stack to keep a record of the points for determining the convex hull [65]. As the algorithm continues to work on each point, the concavities along the boundary are removed to form a convex hull. The algorithm is described in the following two stages (Figure 2):

Stage 1: First, the bottom-most point must be identified (line 5 in Algorithm 3). Then, all the other points are sorted by polar angle in a counterclockwise order on the basis of the bottom-most point (line 6); thus, the sorting of points forms a closed path.

Stage 2: The next stage is to iterate the path and remove all the concave points in counter-clockwise order. During the iteration, three points are selected and checked whether their orientation is counter-clockwise. If not, then the middle point is removed, and the process continues (lines 10–15). At the end of stage 2, a convex hull is created.

As Graham’s scan algorithm [64] returns the convex hull’s points, the instep girth of the foot is computed using Euclidean distance [66] (lines 14–18 in Algorithm 2).

## 4. Application Description and Implementation

### 4.1. Application Architecture

**Figure 3:**
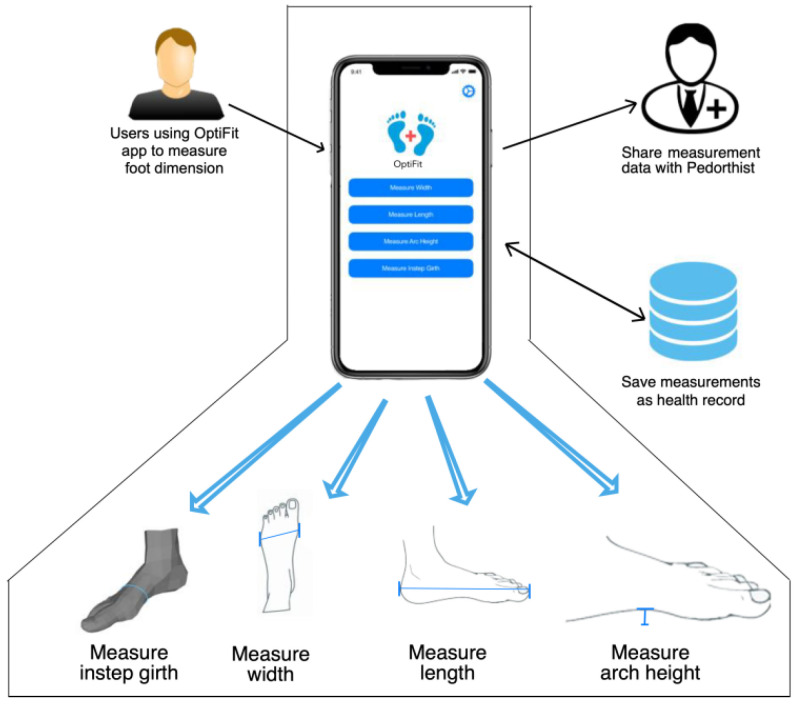
Application architecture.

The core components of the application architecture are illustrated in Figure 3. The primary goal of the application is to simplify the process of foot measurement as much as possible. Users capture images of a foot with a coin (as a reference object) using the application for measuring foot length, width, and arch height. To measure the instep girth, users provide a 3D mesh of their foot that can be generated using the structure sensor (however, the application is also compatible with LiDAR) connected with their devices. Moreover, an OBJ file of the 3D model can be uploaded through media access. Lastly, the calculated values are saved locally to keep a record of all the measurements taken. A sharing option is available in the application that allows for the users to share the measurements with a pedorthist.

### 4.2. Application Settings Activity

**Figure 4:**
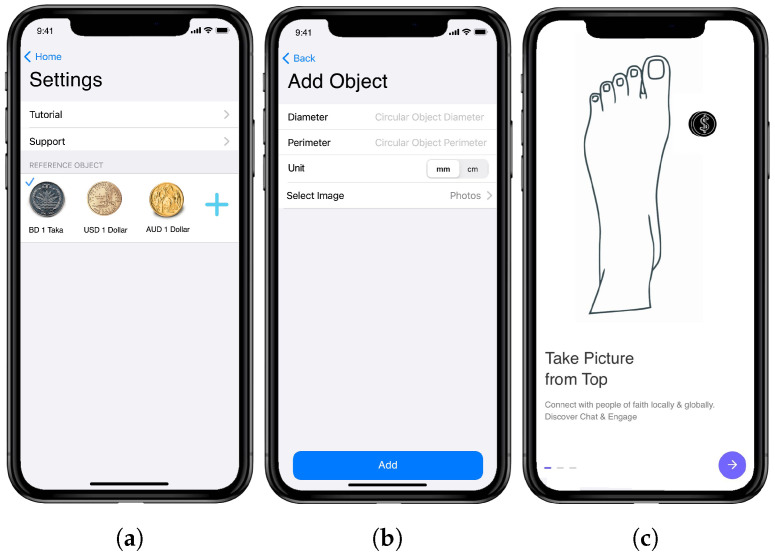
Application settings: (**a**) main screen; (**b**) adding reference object; (**c**) tutorial screen.

Figure 4 shows several screenshots of our application settings. The application includes a tutorial for users on how to measure the dimensions (see Figure 4c). In addition, an email option is provided for the users, so that any bug or problem associated with the application can be reported (see the support option in Figure 4a). A circular reference object is required for the measurement, and users can not only choose reference objects from a list of predefined coins, but also add their own objects (see Figure 4a). The reference object is essential because it provides the unit of the measurement and the calibration accuracy to the users regarding the precision of the measurement. Users must provide two parameters, diameter and perimeter, to the application if they choose to add their own reference objects (see Figure 4b). The diameter of the object is used to determine the pixel-per-metric unit; eventually, the application calculates the perimeter of the object. Then, the application compares the calculated and actual perimeters to determine calibration accuracy.

### 4.3. Foot Length, Width, and Arch Height Measurement

**Figure 5:**
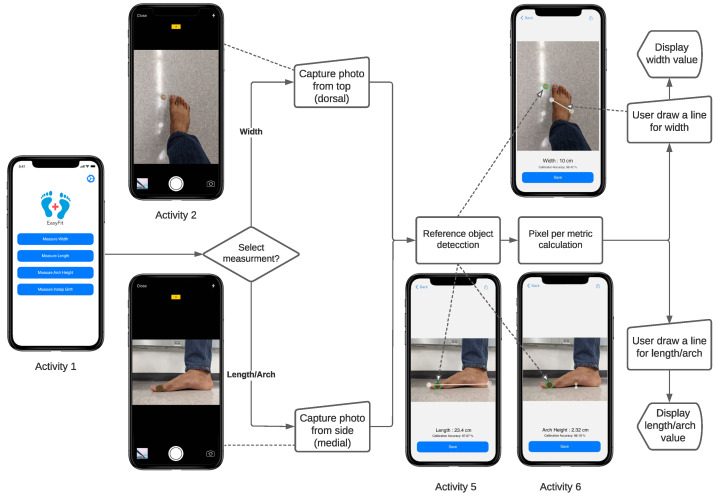
Activity diagram for the measurements of length, width, and arch height.

The three straightforward measurements, namely, length, width, and arch height, are calculated from a 2D image using the application. Users must capture an image of the foot with the reference object using a smartphone camera. Afterward, the measurements are computed and displayed on the next activity. The steps are described as follows according to Figure 5:

1. On the home screen (Activity 1), the users must select their desired dimension from four options (length, width, arch height, and instep girth).
2. For the width measurement, the users must capture the image from the top view (Activity 2), and for the length and the arch height measurement, the users must capture the image from the side view (Activity 4).
3. When the users capture an image with a reference object, Hough transform [63] is applied to the image, and the circular reference object, which was a coin in our case, is detected. This occurs for the measurement of each dimension: length, width, and arch height (Activities 3 + 5 + 6).
4. While the coin is detected, the total number of pixels along its diameter is calculated.
5. The unit, the pixel-per-metric value, is computed by dividing the total number of pixels by the actual diameter of the coin (Algorithm 1).
6. The users must select and move a line that appears on the screen. The purpose of the line is to provide two endpoints of a foot dimension from the image. The distance between these two points is shown on the screen interactively and can be stretched by dragging its endpoints. According to the selected option (length, width, or arch height), the users must move the line to the appropriate position.
7. As the total number of pixels between the two endpoints of the line is obtained, it is divided by the pixel per metric constant to obtain the final measurement of the selected foot dimension (Activities 3 + 5 + 6).

### 4.4. Instep Girth Measurement

**Figure 6:**
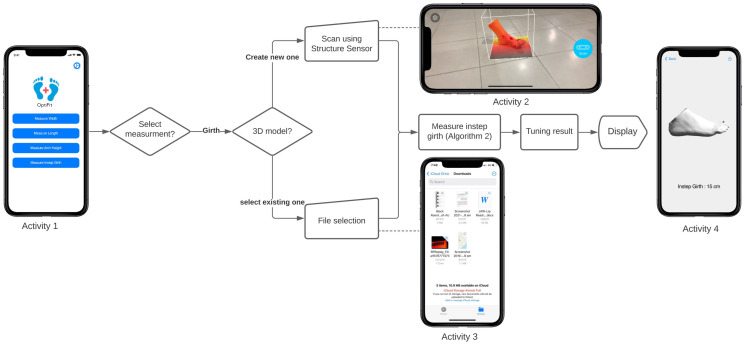
Activity diagram for the instep girth measurement.

**Figure 7:**
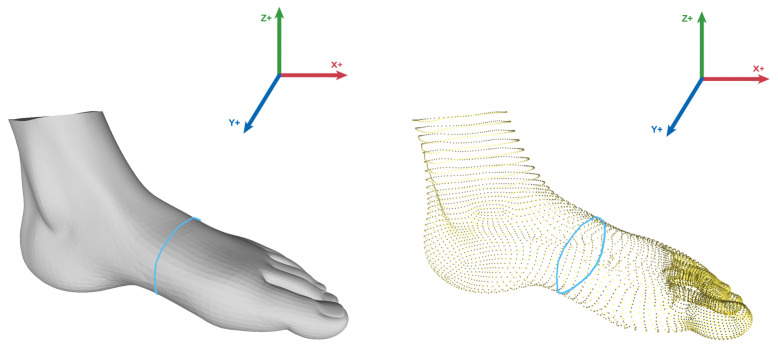
Instep girth measurement from 3D mesh.

To measure the instep girth, the users need a structure sensor (or LiDAR technology) to create a 3D mesh of the foot. The users can also select an OBJ file from the smartphone’s local storage using a file picker, which takes a couple of seconds to process and finally shows the calculated result on the screen. The steps of measuring the dimension are described below according to Figure 6:

1. After selecting the girth measurement from the provided options (Activity 1), the users are given two choices on how to provide a 3D mesh of the feet.
2. The users can either use a structure sensor for scanning the feet (Activity 2) or select an OBJ mesh file of the feet from the smartphone’s local storage (Activity 3).
3. The application fetches all the coordinate points of the foot mesh.
4. As we must measure the middle circular region of the foot (Figure 7), the *x* coordinates should be sorted to obtain its middle point. We achieved this by sorting the coordinate points on the basis of the *x*-axis, taking the sum of the first and last coordinate value, and eventually dividing the sum by 2. Then, only those *y* and *z* coordinates that have the values of *x* coordinates within a specific range are considered. This approach provides a 2D set of coordinates (Algorithm 2).
5. After filtering out the redundant coordinates, we applied Graham’s scan algorithm [64] (Algorithm 3) to determine the convex hull of the points. The idea of Graham’s scan algorithm is simple; it starts from the bottom-most point (or point with the minimal *y* coordinate) and continues wrapping the points in a counter-clockwise direction; this procedure eliminates the irrelevant coordinates and prepares a convex hull [67].
6. Lastly, the instep girth is calculated as the sum of the Euclidean distances between the two neighboring points along the convex hull and displayed on the screen (Activity 4).

## 5. Experimental Evaluation and Discussion

We evaluated *OptiFit*’s accuracy by using it to measure 19 medical-grade silicon foot models. As the reliability of the manual measurement (MM) is relatively high, we compared the application-based measurement (AM) with MM. We provide a descriptive statistical comparison, and a significant discrepancy was noticed between MM and AM in the paired *t*-test for the instep girth. We derived a regression model and demonstrate how the model effectively reduces the instep girth measurement error/discrepancy. Lastly, we present the accuracy and the relative error analysis of four-foot measurements for the two methods (MM and AM).

### 5.1. Foot Measurement Data

In this study, we used 19 medical-grade silicon foot models (12 males and 7 females). The minimal and maximal ages of those foot models were 10 and 65 years old, respectively. The reason to use 10 as the minimal age is that it is very uncommon to need a custom shoe for a child below that age. We categorized those models into three groups on the basis of age: Group 1 (10–16 years), Group 2 (17–35 years), and Group 3 (36–65 years).

Initially, our two coinvestigator pedorthists individually measured the length, width, arch height, and instep girth of the foot models manually using a measuring tape and sliding calliper. If the variations in their measurements were less than 5%, the average was taken; otherwise, they discussed among themselves and came to a consensus on the measurement value, which is referred to as the MM data. Afterward, both pedorthists individually used the *OptiFit* application to measure the length, width, arch height, and instep girth of all foot models. In our study, we used the mean of the two individuals’ measurements, which is referred to as the AM data. To prevent the ordering effect, evaluators randomly choose the order of measurement, i.e., app-based measurement is followed by manual measurement and vice versa.

### 5.2. Statistical Comparison between MM and AM

Different statistical approaches, namely, the maximal, minimal, mean, and standard deviation of the four foot measurements based on different age groups are shown in Table 2.

Table 2 shows that the dataset had significant variance in the foot dimensions. Additionally, Table 3 indicates the differences in the maximal, minimal, mean, and standard deviation between AM and MM (AM–MM). The difference was positive when AM is larger than MM; otherwise, the difference was negative. The difference was either positive or negative in length, width, and arch height measurement, as they are heavily dependent on selecting the two end points. AM was always larger than MM in the instep girth measurements for all age groups.

### 5.3. Measurement Method Effect (*p*-Value) on MM and AM

Table 2 and Table 3 indicate that there was a difference between AM and MM of the instep girth for the categorized groups. A two-tailed paired *t*-test was conducted, which is shown in Table 4. In our context, the paired *t*-test is used to test whether the mean difference between the pairs of MM and AM is zero. The statistical significance level was set to 0.05. Results of the paired *t*-test indicate that there was a nonsignificant medium difference between MM and AM for length (*p* = 0.2925), width (*p* = 0.8125), and arch height (*p* = 0.502). Thus, the two methods were not different in three out of the four foot dimension measurements, except instep girth (*p* = 1.132 × 10^−12^).

### 5.4. Tuning AM of the Instep Girth by Linear Regression Model

**Figure 8:**
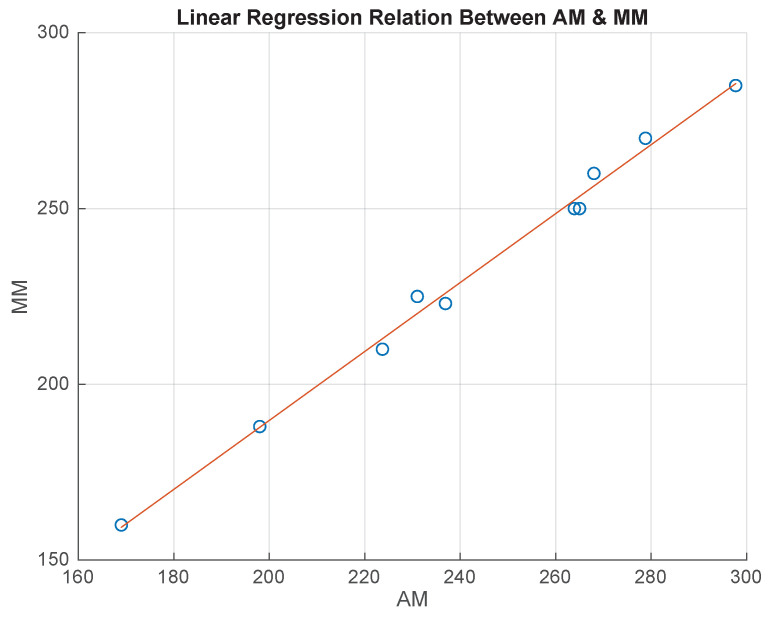
Linear regression between AM and MM for the instep girth of the training dataset.

As the AM had a significant discrepancy with respect to MM regarding the instep girth dimension, a linear regression model was implemented to understand whether this error was systematic. For this purpose, the 19 medical-grade silicon foot models were categorized into 10 foot models for the training dataset and 9 foot models for the test dataset, while taking models from each group. Using the training dataset, a linear regression, MM = a×AM + b, was formed to adjust the AM to move it closer to MM (Figure 8). Table 5 displays that the formation of linear regression had a high *R*${}^{2}$ value of 0.9939, suggesting that the systematic error could be used to adjust the AM to generate the specified MM. The test dataset was applied to validate the adjusted AM result with respect to the MM. Moreover, a two-tailed paired *t*-test was conducted on the test set, and there was no difference between the two measurement methods (*p* = 0.3361) (Table 6).

### 5.5. Accuracy and Relative Error Analysis

**Figure 9:**
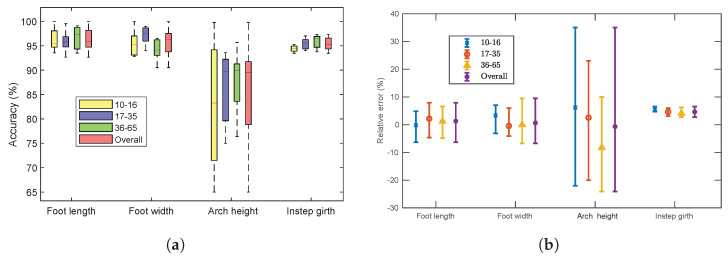
Accuracy and relative error for different age groups: (**a**) accuracy of four foot measurements; (**b**) mean and its standard error (error bar) of relative difference (%) between MM and AM.

Figure 9 shows the accuracy and the relative error of the foot measurements. The overall accuracy of foot length, width, arch height and instep girth was 95.23%, 96.54%, 89.14%, and 95.27%, respectively (shown in Figure 9a).

**Figure 10:**
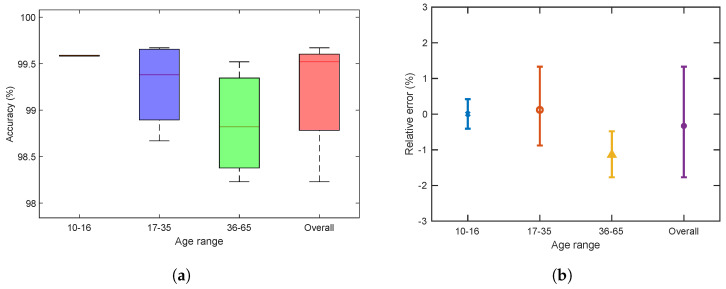
Accuracy and relative error for the instep girth measurement of different age groups after applying linear regression: (**a**) accuracy; (**b**) mean and its standard error (error bar) of relative difference (%) between MM and AM.

The accuracies were above 90% for the foot dimension measurements except for the arch height. The accuracy of the arch height notably varied between all age groups, particularly for Group 1. Because this dimension is extremely small to measure, it often becomes inflexible to estimate for a small foot. The accuracy of the instep girth was also represented before applying the linear regression model (Figure 9a). Although the accuracy of the instep girth was above 90%, we adjusted the calculation. According to domain experts, this specific dimension significantly helps in measuring the whole structure of a foot, and a modest discrepancy may reduce that possibility [45,67]. Therefore, we applied a linear regression model using a training dataset for the instep girth, and the overall accuracy increased by 4.25% (i.e., from 95.27% to 99.52%) while applying it to the test set (Figure 10a). However, Figure 9b shows the relative errors for the four foot dimensions. The mean relative errors were generally between −10% and +10%, except for the dimensions of arch height. The relative error of the arch height is comparatively higher (from −25% to +35%) than that of other measurements. Figure 10b illustrates that the relative error of the instep girth was between −2% and 1.5% after applying the adjustment method. The relative error between the MM and the AM for each dimension was calculated using the following equation:

(3) $$error\;\left(\%\right)=\frac{AM-MM}{MM}\times 100\%$$

Measuring the foot width using the application is more straightforward than other measurements are owing to the visibility of the maximal horizontal breadth. In addition, foot length, width, and arch height measurements are dependent on the calibration accuracy of a coin or a circular object. The difference between MM and AM for these dimension measurements can be optimized by precisely finding the identical end points. We did not apply linear regression to foot length, width, and arch height measurements, and these computations using the application are easily repeatable and reproducible. Conversely, a linear regression model was implemented to adjust the AM of the instep girth only because this dimension relies on the 3D mesh of a foot.

## 6. Conclusions and Future Work

This study introduced a smartphone iOS application that measures four primary foot dimensions: length, width, arch height, and instep girth. To evaluate the application, we used 19 medical-grade silicon foot models and measured the specified dimensions of those models through both manual and application-based approaches. Our experimental results demonstrate that the performance of the application was satisfactory. In addition, the study highlighted the significant differences between the MM and the AM of instep girth and established a solution for adjusting the error. However, good definitions and registration procedures are important for both measurement methods. To measure the instep girth, a 3D scanned model of the foot is required. Hence, it is possible that the participant’s fatigue can make the shape change. Moreover, locating the critical points, by definition, is not always easy. For example, arch height varies significantly from person to person, and this dimension is too small to be measured. However, the huge advantage is that our developed application’s procedure is easily repeatable and reproducible. The important fact is that the registration, dimension definition, and used algorithm play the most vital role in any kind of measurement. Therefore, our smartphone solution for measuring the foot dimensions can be a leading method in the custom footwear industry because of the cost, precision of the algorithm, and the allowable tolerances for each measurement.

In the future, we plan to evaluate *OptiFit* using real feet. We also plan to greatly facilitate the entire process of measuring the foot dimensions through augmented reality (AR), which analyzes the environment and can perform accurate depth estimations of the surroundings. Additionally, AR would remove the need for using a reference object and would easily create a 3D mesh of the foot for measuring the instep girth dimension. All these aspects unlock many new opportunities to render the experience of measuring foot dimensions easier and faster for custom footwear. 

## Figures and Tables

**Table 1:** Application review summary and comparison.

App Name	Length	Width	Arch Height	Instep Girth
MOBILE FOOT ID	✓	✓	✕	✕
FitMyFoot	✓	✓	✕	✕
Feet Meter	✓	✓	✕	✕
Nimco Professional Shoe Sizing	✓	✓	✕	✓
Fischer Scan-Fit	✓	✓	✓	✕
3D Avatar Feet	✓	✓	✕	✓
3DSizeMe	✓	✓	✕	✕
*OptiFit* (This study)	✓	✓	✓	✓

**Table 2:** Descriptive statistics of all measurements obtained from the two methods, MM and AM. All measurements are in millimetres (mm).

Foot Measurements	Age Group (Years)	Max (mm)		Min (mm)		Mean (mm)		Std. Dev. (mm)	
		MM	AM	MM	AM	MM	AM	MM	AM
Foot length	Group 1 (10–16)	231.78	236.25	160.02	149.86	200.35	196.10	29.77	35.88
	Group 2 (17–35)	279.40	281.43	233.68	240.03	257.88	262.94	14.84	16.89
	Group 3 (36–65)	236.22	248.92	177.80	169.16	220.45	223.99	23.36	31.64
	All	279.40	281.43	160.02	149.86	233.95	236.57	31.55	37.20
Foot width	Group 1	95.25	95.20	76.20	78.74	83.82	85.12	8.10	7.19
	Group 2	106.68	113.11	93.98	90.12	101.02	100.80	4.06	6.68
	Group 3	101.60	95.50	78.74	73.41	89.11	87.86	7.97	7.62
	All	106.68	113.11	76.20	73.41	93.64	93.41	9.56	9.87
Arch height	Group 1	50.80	50.90	12.70	17.15	32.39	32.23	16.11	13.95
	Group 2	63.50	62.48	45.72	40.64	50.48	51.45	5.67	7.51
	Group 3	63.50	58.17	30.48	24.92	44.03	39.63	11.77	12.13
	All	63.50	62.48	12.70	17.15	44.31	43.24	12.30	12.87
Instep girth	Group 1	220	231.8	160	169	198.75	209.8	26.58	28.08
	Group 2	285	297.70	230	239	256.67	268.4	15	15.30
	Group 3	274	284.6	188	198	234.17	243.5	32.54	32.64
	All	285	297.70	160	169	237.37	248.2	32.14	32.72

**Table 3:** Difference between the application-based measurement (AM) and the manual measurement (MM) of all foot dimensions. All measurements are in millimetres (mm).

Foot Measurements	Age Group (Years)	AM-MM (Millimetre)			
		Max	Min	Mean	Std. dev.
Foot length	Group 1 (10–16)	4.47	−10.16	−4.25	6.11
	Group 2 (17–35)	2.03	6.35	5.06	2.05
	Group 3 (36–65)	12.70	−8.64	3.54	8.28
	All	2.03	−10.16	2.62	5.65
Foot width	Group 1	−0.05	−2.54	−1.3	−0.91
	Group 2	6.43	−3.86	−0.23	2.61
	Group 3	−6.10	−5.33	−1.25	−0.35
	All	6.43	−2.79	−0.23	0.31
Arch height	Group 1	0.10	4.45	−0.16	−2.17
	Group 2	−1.02	−5.08	0.97	1.84
	Group 3	−5.33	−5.56	−4.40	0.36
	All	−1.02	4.45	−1.07	0.58
Instep girth	Group 1	11.80	9	11.05	1.51
	Group 2	12.70	9	11.73	0.30
	Group 3	10.60	10	9.33	0.01
	All	12.70	9	10.83	0.58

**Table 4:** Paired *t*-test for comparing two measurement methods (MM, AM) on foot measurements.

Foot Measurements	T-Score	Measurement Method Effect (*p*-Value)
Foot length	1.0844	0.2925
Foot width	−0.2408	0.8125
Arch height	−0.6859	0.5020
Instep girth	17.3236 *	<0.0001

* indicates significant at 5% level of significance.

**Table 5:** Linear regression, MM = a× AM + b for the instep girth that had significant differences between AM and MM of the training dataset.

Foot Measurement	Linear Regression Equation	$R^{2}$
Instep girth	MM = 0.98048 × AM − 6.35234	0.9939

**Table 6:** Paired *t*-test for comparing the two measurement methods (MM, AM) of the instep girth measurement using the test dataset after applying linear regression.

Foot Measurement	T-Score	Measurement Method Effect (*p*-Value)
Instep girth	−1.023	0.3361

## Data Availability

Not applicable.
